# Tissue Distribution, Histopathological and Genotoxic Effects of Magnetite Nanoparticles on Ehrlich Solid Carcinoma

**DOI:** 10.1007/s12011-022-03102-z

**Published:** 2022-01-15

**Authors:** Heba Bassiony, Akmal A. El-Ghor, Taher A. Salaheldin, Salwa Sabet, Mona M. Mohamed

**Affiliations:** 1 Department of Zoology, Faculty of Science, Cairo University, Giza, 12613 Egypt; 2 Pharmaceutical Research Institute, Albany College of Pharmacy and Health Sciences, Albany, NY USA; 3 Director of Biotechnology Program, Faculty of Science, Galala University, Suez, 43511 Egypt

**Keywords:** Magnetite nanoparticles, Histology, Ehrlich tumor, DNA damage, Mice

## Abstract

Nanoparticles can potentially cause adverse effects on cellular and molecular level. The present study aimed to investigate the histopathological changes and DNA damage effects of magnetite nanoparticles (MNPs) on female albino mice model with Ehrlich solid carcinoma (ESC). Magnetite nanoparticles coated with L-ascorbic acid (size ~ 25.0 nm) were synthesized and characterized. Mice were treated with MNPs day by day, intraperitoneally (IP), intramuscularly (IM), or intratumorally (IT). Autopsy samples were taken from the solid tumor, thigh muscle, liver, kidney, lung, spleen, and brain for assessment of iron content, histopathological examination, and genotoxicity using comet assay. The liver, spleen, lung, and heart had significantly higher iron content in groups treated IP. On the other hand, tumor, muscles, and the liver had significantly higher iron content in groups treated IT. MNPs induced a significant DNA damage in IT treated ESC. While a significant DNA damage was detected in the liver of the IP treated group, but no significant DNA damage could be detected in the brain. Histopathological findings in ESC revealed a marked tumor necrosis, 50% in group injected IT but 40% in group injected IP and 20% only in untreated tumors. Other findings include inflammatory cell infiltration, dilatation, and congestion of blood vessels of different organs of treated groups in addition to appearance of metastatic cancer cells in the liver of non-treated tumor group. MNPs could have an antitumor effect but it is recommended to be injected intratumorally to be directed to the tumor tissues and reduce its adverse effects on healthy tissues.

## Introduction

Toxic effects of nanoparticles depend on their exposure route, exposure duration, and physicochemical properties such as size, shape, reactivity, and material composition [1, 2]. Studies conducted on iron oxide nanoparticles had controversial results. For instance, a study suggested non-toxicity of iron oxide nanoparticles under in vivo condition whereas others reported minimal toxicity or severe cell death [3–5]. Magnetite nanoparticles (MNPs) have been used in biomedical applications including delivery of drugs or genes, labeling of macromolecules and cells, tissue engineering, magnetic transfection, chelation therapy, and in the destruction of tumor tissue through hyperthermia [6–8]. Therefore, evaluation of the health impact of iron oxide nanoparticles is important [9]. Several studies have reported MNP-induced cytotoxicity. MNPs showed toxicity on rat liver-derived cell line (BRL3A) at high concentration. Moreover, super paramagnetic iron oxide NPs caused toxic effect with repeated injections in rats, rabbits, dogs, and monkeys [10]. Dextran-MNPs and uncoated MNPs caused cell death and reduced proliferation of fibroblasts in vitro [11]. Another study demonstrated that iron oxide NPs caused moderate cytotoxicity on Vero cell line [12].

MNPs caused hyperthermia-mediated cell death due to oncotic necrosis in a mouse xenograft model of a human head and neck squamous cell carcinoma cell line (Tu212) [13]. Another study showed that MNPs combined with daunorubicin were highly biocompatible and safe nanoparticles, suitable for the treatment of hematologic malignancies and were able to overcome multidrug resistance in mice [14].

Minor variations in histology of both the spleen and liver were observed at high concentrations, i.e., 200 × higher doses than that used for MR imaging [15, 16]. Single intra-tracheal instillation of MNPs in mice caused chronic inflammatory responses through microgranulomatous changes in the alveolar space [17]. However, rats exposed to single intratracheal instillation of MNPs revealed no abnormalities in the liver, kidneys, and spleen, whereas the lungs developed a weak pulmonary fibrosis. As well as MNPs caused injury of cell membranes of both animal and human lung cells that became irregular and lost continuity, and finally, the cells were fragmented [12, 18].

Intravenous injection of MNPs stabilized by 2, 3-dimercaptosuccinic acid (DMSA) in mice formed clusters of MNPs in blood vessels, increased number of leukocytes in the organs, and induced pulmonary fibrosis in the lung. After 90 days of injection, lung parenchyma became normal, except for a few cells that contained MNPs and small groups of inflammatory cells [19].

In addition, nanoparticles can gain access to the nucleus and induce genotoxic effect [20]. Swiss mice, treated with MNPs coated with polyaspartic acid, showed increase in micronucleus frequency [21, 22].

The present study aimed to investigate the histopathological effects and induction of DNA damage by MNPs coated with L-ascorbic acid on ESC and normal tissue that may give insight into the toxicological mechanism of application of nanoparticles. Also, this study aimed to assess the tissue distribution of MNPs in ESC bearing mice.

## Materials and Methods

### Preparation and Characterization of Magnetite Nanoparticles (MNPs)

MNPs capped with L-ascorbic acid with 25.0 ± 5.0 nm size were synthesized by co-precipitation. In this method, 0.25 g of FeCl_3_ anhydrous was dissolved in 25 mL sterile saline. Then, 10 mL of 6 M Na_2_CO_3_ was added drop by drop with continued stirring for 10 min; the solution turned brown color. Then 0.12 g ascorbic acid was added with vigorous stirring for 15 min; the color of solution turned black. Finally, the solution was completed to 50 mL with sterile saline and sterilized by autoclaving [23]. Physicochemical properties of MNPs were characterized using High-Resolution Transmission Electron Microscope (HR-TEM, FEI, Tecnia G20), Particle size analyzer (Malvern, ZS), and X-ray Diffraction (XRD, PanAnalytical, X’pert Pro) [24, 25].

### Mice Treatment

Study protocol was approved by the Institutional Animal Care and Use Committee (IACUC), Faculty of Science, Cairo University, Egypt (approval number: CUFS/ F/ Cell Biol./ 02/ 13). All the experimental procedures were carried out in accordance with international guidelines for care and use of laboratory animals as described before [23, 26].

Six-week-old female Swiss albino mice with body weight 25–30 g were obtained from animal house of National Cancer Institute, Cairo University, Egypt. Upon arrival, mice were randomly transferred to plastic cages containing sawdust bedding and allowed to acclimatize for 2 weeks before the start of the experiment. They were housed under the standard conditions of room temperature (22–24 °C), humidity (45–65%), light (12 h light/12 h dark cycles), and received food and tap water ad libitum.

Ehrlich ascites carcinoma bearing mouse was obtained from National Cancer Institute (Cairo, Egypt). Viability of cells was estimated by staining with Trypan blue dye and counting with Neubauer hemocytometer, as previously described by Dagli et al., 1992 [27, 28]. Mice were randomly divided into six groups (six mice/group), control untreated, IP MNPs, IM MNPs, ESC, ESC + IP MNPs, and ESC + IT MNPs (where IP = intraperitoneally injected with MNPs, IM = intramuscularly injected with MNPs, and IT = intratumorally injected with MNPs).

For tumor induction, mice of groups 4, 5, and 6 were implanted with 0.2 mL of Ehrlich tumor cell suspension (containing about 2 × 10^6^ viable cells) IM in the thigh of the left hind leg. Once solid tumor appeared on the day 14 (time taken for detectable mass of tumor to appear depends on the amount and viability of the injected cells, mostly 12–14 days after initial injection), mice in groups 2, 3, 5, and 6 were injected with 60 mg/kg of MNPs day by day for fourteen injections. Groups that were not injected with MNPs were injected with saline (groups 1 and 4). Finally, animals were sacrificed by cervical dislocation after being anesthetized using sodium thiopental (0.5%). Autopsy samples were taken from the tumor, thigh muscle, liver, kidney, lung, spleen, and brain for subsequent analyses. Part of the samples was preserved in 10% formaldehyde for histopathological examination, while another part was preserved in –80 °C for assessment of iron content, and comet assay.

### Measurement of Iron Bio-Distribution

Tissue samples from the tumor, thigh muscle, liver, kidney, lungs, spleen, heart, and brain weighing 100–900 mg (mean 300 ± 20 mg) were dried in the muffle to form ash. The ash was digested with concentrated hydrochloric acid, then was diluted with distilled water and was filtered to get rid of impurities. Iron concentration was estimated using inductively coupled plasma optical emission spectrometry ICP-OES (Thermo Scientific™ iCAP™ 7000) [29, 30]. The amount of iron was calculated from the linear portion of the generated standard curve [31].

### Histological Analysis

Samples were taken from the ESC, liver, kidney, lung, spleen, and brain and were fixed in 10% formal saline for 24 h. Then were dehydrated in ascending series of alcohol, cleared in xylene, and embedded in paraffin wax at 56 °C for 24 h. Paraffin sections of 4-µm thickness were collected on glass slides, dewaxed in xylene, hydrated in descending series of alcohol, stained by hematoxylin and eosin stains, dehydrated in ascending series of ethyl alcohol, cleared in two changes of xylene, and mounted with DPX. Finally, slides were examined by light electric microscope (Olympus, CX41, Japan).

### DNA Damage Analysis by Comet Assay

DNA damaging effects of MNPs on tumor, muscle, liver, and brain tissues were investigated using the single cell gel electrophoresis (SCGE) or called alkaline comet assay [32]. Slides were prepared using 10 µl of cell suspension and 70 µl of 0.5% low melting agarose (Thermo scientific, USA) and spread on a fully frosted slide pre-dipped in 1% normal melting agarose. Slides were incubated in cold lysis buffer (2.5 M NaCl, 100 mM EDTA, and 10 mM Trizma base, pH 10, with freshly added 1% Triton X-100 and 10% DMSO 30 min before use) for 24 h at 4 °C in darkness. Slides were washed for 10 min in deionized H_2_O, incubated for 20 min in electrophoresis alkaline buffer (300 mM NaOH and 1.0 mM EDTA, pH 13) to allow unwinding of the DNA and electrophoresed for 20 min at 300 mA and 25 V (0.90 V/cm). Then slides were neutralized in 0.4 M Tris, pH 7.5, fixed in 100% cold ethanol, and stained with ethidium bromide (0.2 µg/mL). The comets were observed using Nikon epifluorescence microscope (filter B-3A; excitation: *λ* = 420 − 490 nm; emission: *λ* = 520 nm) at a magnification of 400 × . For each sample, 50 cells were scored using Comet 5 image analysis software developed by Kinetic Imaging, Ltd. (Liverpool, UK).

### Statistical Analysis

Data were analyzed using statistical package for the social sciences software (SPSS) version 20.0. Student’s *t*-test or analysis of variance (ANOVA) was used to illustrate the comparison of iron distribution, percentage of DNA in comet tail in different tissues among groups and followed by Duncan multiple comparison test to determine differences among groups. The results are presented as mean ± standard error (SE) for three samples for each group. *P*-value < 0.05 was considered as statistical significance.

## Results

### Properties of MNPs

**Fig. 1:**
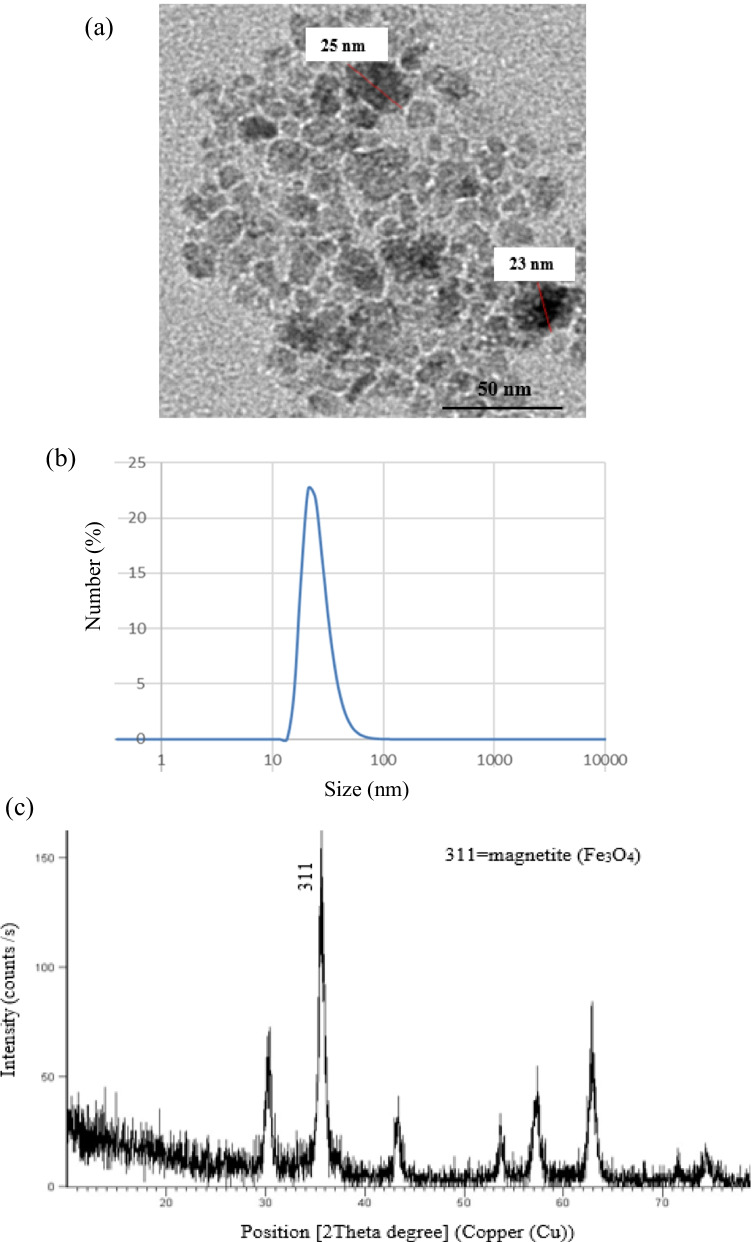
Characterization of synthesized magnetite nanoparticles. **a** HR-TEM image of the spherical shape of MNPs with an average size of 25.0 ± 5.0 nm. **b** Particle size distribution by number measured by dynamic light scattering (DLS) shows the size of MNP is 25.8 nm from the observed peak. **c** XRD diffraction pattern shows the formation of Fe_3_O_4_

Physicochemical properties of synthesized MNPs were characterized via different techniques as have been described before [23]. As shown in Fig. 1, HR-TEM revealed that synthesized MNPs have homogeneous size 25.0 ± 5.0 nm, spherical in shape, and loosely agglomerated with single-crystal. Particle size distribution obtained by dynamic light scattering (DLS) showed that the hydrodynamic average size of MNPs is 25.8 nm. XRD phase analysis confirmed the phase formation of Fe3O4 crystal.

### Iron Bio-Distribution in Different Organs

**Fig. 2:**
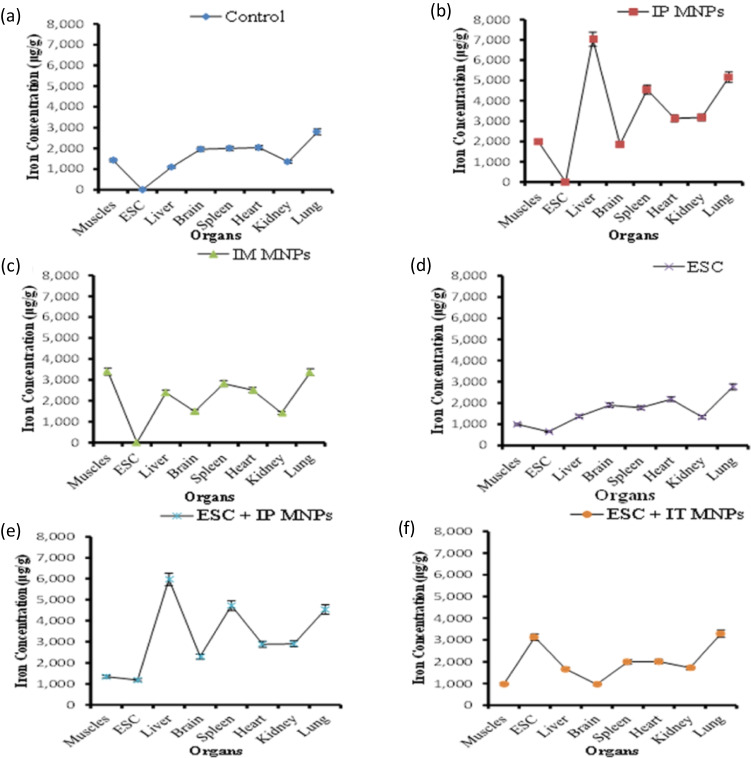
Comparison of iron distribution in different organs in each mice group. **a** Control group, **b** IP MNP group, **c** IM MNP group, **d** ESC group, **e** ESC + IP MNP group, and **f** ESC + IT MNP group. Each point represents the mean of iron content in µg/g

Measurements of iron concentrations in micrograms per gram of dry organ weight of triplet samples are presented as mean ± standard error of mean (SE) in Table 1 and presented in Fig. 2.

**Table 1:** Comparison of iron distribution (µg/g) in different organs among groups

Group Tissue	1 (control)	2 (IP MNPs)	3 (IM MNPs)	4 (ESC)	5 (ESC + IP MNPs)	6 (ESC + IT MNPs)
Tumor				650.8 ± 39.6 ^a^,*	1182.6 ± 76.6 ^b^	3120.5 ± 68.7 ^c^
Muscles	1426.8 ± 77.7 ^a^	1981.6 ± 33.4 ^b^	6395.4 ± 110.8^c^,*	990.7 ± 62.2 ^a^	1343.2 ± 80.1^b^	972.7 ± 24.8 ^a^
Liver	1093.1 ± 56.9 ^a^	6032.3 ± 95.6^c^,*	2387.28 ± 86.4 ^b^,*	1369.7 ± 20.0 ^a^	5972.46 ± 61.2 ^c^,*	1663.5 ± 95.1^a,b^,*
Brain	1960.6 ± 68.1 ^b^	1954.4 ± 49.38 ^b^	1491.6 ± 88.4 ^a,b^	1899.4 ± 91.6 ^b^	2161.9 ± 128.67 ^b^	966.8 ± 73.4 ^a^
Spleen	2001.4 ± 75.4 ^a^	4542.1 ± 107.3^b^,*	2820.8 ± 54.4^a^	1779.7 ± 84.3 ^a^	4719.4 ± 72.9 ^b^,*	2000.0 ± 56.4 ^a^
Heart	2042.1 ± 105.9^a^	3131.7 ± 108.6 ^b^,*	2510.3 ± 131.7 ^a,b^	2177.8 ± 112.6 ^a^	3074.5 ± 110.9^b^,*	2011.1 ± 97.8^a^
Kidney	1350.0 ± 66.8 ^a^	3173.6 ± 111.8 ^b^	1416.7 ± 85.1^a^	1342.4 ± 125.6^a^	2897.8 ± 120.6 ^b^	1726.9 ± 62.4^a^
Lung	2798.1 ± 87.5 ^a^	5158.4 ± 39.0 ^c^,*	3362.5 ± 76.5 ^b^	2764.6 ± 86.3 ^a^	4530.42 ± 95.7^c^,*	3286.9 ± 7.4 ^b^

All values are represented as mean ± standard error of mean (SE). There is a significant difference between groups by using one-way ANOVA at *P* < 0.05 followed by Duncan multiple comparison test. The same letter means that there is no significant difference between the two groups by using Duncan multiple comparison test (*P* > 0.05). The different letters mean that there is a significant difference between the two groups by using Duncan multiple comparison test (*P* < 0.05). *Statistically significant compared with negative control using Student’s *t*-test

As shown, mice groups treated IP with MNPs and IP with MNPs + ESC have highest accumulation of iron in their organs in comparison to other groups. The highest iron concentrations of these groups were detected in the liver (5972.46 ± 61.2 and 7032.3 ± 108.6 µg/g, respectively), spleen (4542.1 ± 107.3 and 4719.4 ± 72.9 µg/g, respectively), and lung (4530.42 ± 149.6 and 5158.4 ± 39.0 µg/g, respectively).

On the other hand, groups treated locally (IT and IM) with MNPs have highest iron concentration in their ESC and muscles. There is no significant difference in iron concentrations in organs of mice treated only with MNPs compared to the control group.

Statistically significant difference was found in the distribution of iron in ESC among groups with highest iron concentration in ESC treated IT with MNPs (3120.5 ± 68.7 µg/g) (Fig. 2f).

In muscles, group treated IM with MNPs showed the highest accumulation of iron compared with other groups (6395.4 ± 110.8 µg/g) (Fig. 2c).

The highest accumulation of iron in liver was found in IP treated groups with MNPs, with no significant difference between them (6032.3 ± 95.6 and 5972.46 ± 61.2 µg/g respectively) but are significantly different from the other groups (Fig. 2b, e).

Similarly, the highest accumulation of iron in the spleen was observed in IP treated groups with MNPs, IP MNPs, and ESC + IP MNPs. They have similar and significantly higher concentration of iron (4719.4 ± 72.9 and 4542.1 ± 107.3, µg/g respectively) (Fig. 2b, e) compared to the control group (2001.4 ± 75.4 µg/g) (Fig. 2a).

IP treated group with MNPs and ESC treated IP with MNPs have significantly higher iron concentration in their brain tissues (3131.7 ± 108.6 and 3074.5 ± 110.9 µg/g respectively) (Fig. 2b, e) compared to the control group (2042.1 ± 105.9 µg/g) (Fig. 2a).

Similarly, lung tissues have the highest concentration of iron in IP treated group with MNPs and ESC treated IP with MNPs (5158.4 ± 39.0 and 4530.4 ± 95.7 µg/g respectively) (Fig. 2b, e). Both have significantly higher iron concentration compared to the control group (Fig. 2a). While the least concentration of iron was found in the control group and mice injected with ESC only (2764.6 ± 86.3 and 2798.1 ± 87.5 µg/g respectively) (Fig. 2a, d).

On the other hand, there was no significant difference in iron concentrations in the brain among groups except for ESC treated IT with MNPs that showed the lowest concentration of iron (966.8 ± 73.4 µg/g) (Fig. 2f).

Finally, for the kidney, the highest concentration of iron was recorded in mice that received IP injection with MNPs (3173.6 ± 111.8 µg/g) (Fig. 2b). In contrast, the least concentration of iron was recorded in mice injected with ESC only (1342.4 ± 125.6 µg/g) (Fig. 2d).

### Histological Examination

**Fig. 3:**
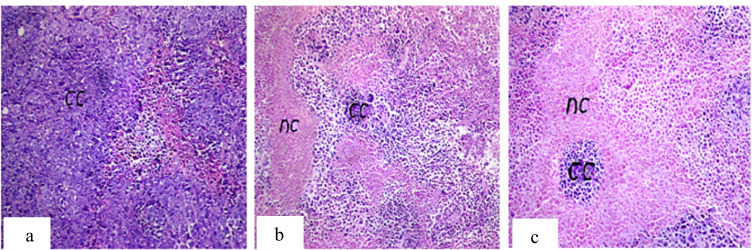
Photomicrograph of ESC of mice. **a** Group 4 showing intact cancer cells (cc) occupying 90% of the skeletal muscle bundles with only 20% necrosis. **b** Group 5 showing 40% necrosis (nc) of the injected Ehrlich tumor cells. **c** Group 6 showing 50% necrosis (nc). H&E, 40 ×

Histopathological examination of ESC shows sheets of small, higher chromatophilic tumor cells of variable shape representing cell proliferation surrounding areas of necrosis and differentiated cells. These tumor cells occupied most of the skeletal muscle bundles and only few areas of necrosis (20%) in non-treated tumor. While necrotic areas significantly increased in tumor groups injected with MNPs, giving that the highest percentage of necrosis was observed in the IT treated group (50%) (Fig. 3).

**Fig. 4:**
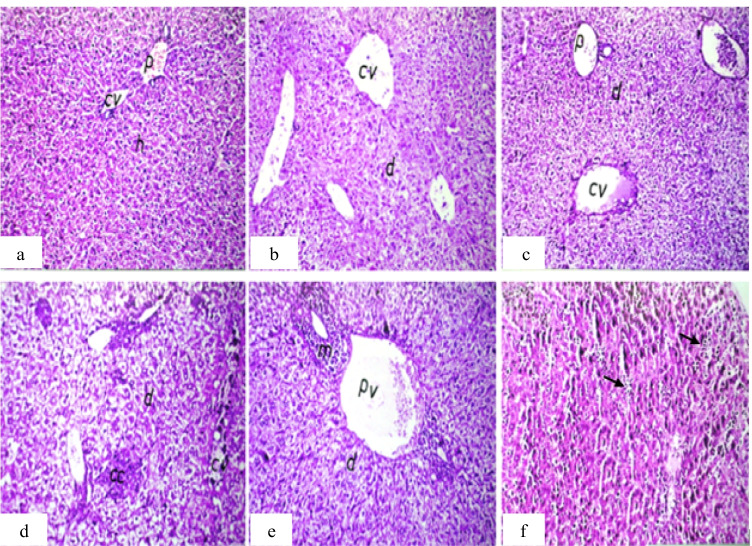
Photomicrograph of liver sections of mice. **a** Group 1 (control) showing normal histological structure of the central vein (cv), portal area (p), and surrounding hepatocytes (h). **b** Group 2 (IP-MNPs) showing dilatation in the central vein (cv) and degeneration in hepatocytes (d). **c** Group 3 (IM-MNPs) showing dilatation and congestion of the central (cv) and portal vein (p) with degeneration in hepatocytes (d). **d** Group 4 (ESC) showing focal metastatic cancer cells (cc) in the degenerated hepatic parenchyma (d) with dilatation in the central vein (cv). **e** Group 5 (ESC + IP-MNPs) showing dilatation in the portal vein (pv) and inflammatory cell infiltration (m) surrounding the bile ducts in portal area and degeneration in hepatocytes (d). **f** Group 6 (ESC + IT-MNPs) showing inflammatory cell infiltration in between hepatocytes (arrow). H&E, 40 ×

For the liver, MNPs induce inflammatory cell infiltration between the hepatocytes and slight congestion of the central and portal vein with hepatocyte degeneration compared to the control group. By comparing the tumor groups, focal metastatic cancer cells in the degenerated hepatic parenchyma were found in non-treated tumor group that did not appear in MNP-treated tumor groups (Fig. 4).

**Fig. 5:**
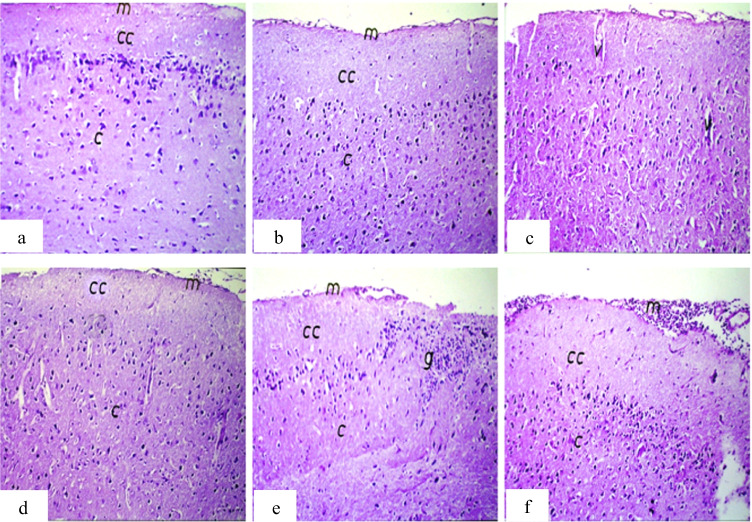
Photomicrograph of brain sections of mice. **a** Group 1 (control) showing normal histological structure of the meninges (m), cerebral cortex (cc), and cerebrum (c). **b** Group 2 (IP-MNPs) showing intact normal histological structure. **c** Group 3 (IM-MNPs) showing mild congestion in the cerebral blood vessels (v). **d** Group 4 (ESC) showing intact normal histological structure. **e** Group 5 (ESC + IP-MNPs) showing focal gliosis (g) in the cerebrum. **f** Group 6 (ESC + IT-MNPs) showing massive number of inflammatory cell infiltration in meninges (m). H&E, 40 ×

For the brain, focal gliosis was observed in the IP treated tumor group which disappeared in the other tumor groups (tumor only and IT treated tumor) (Fig. 5).

**Fig. 6:**
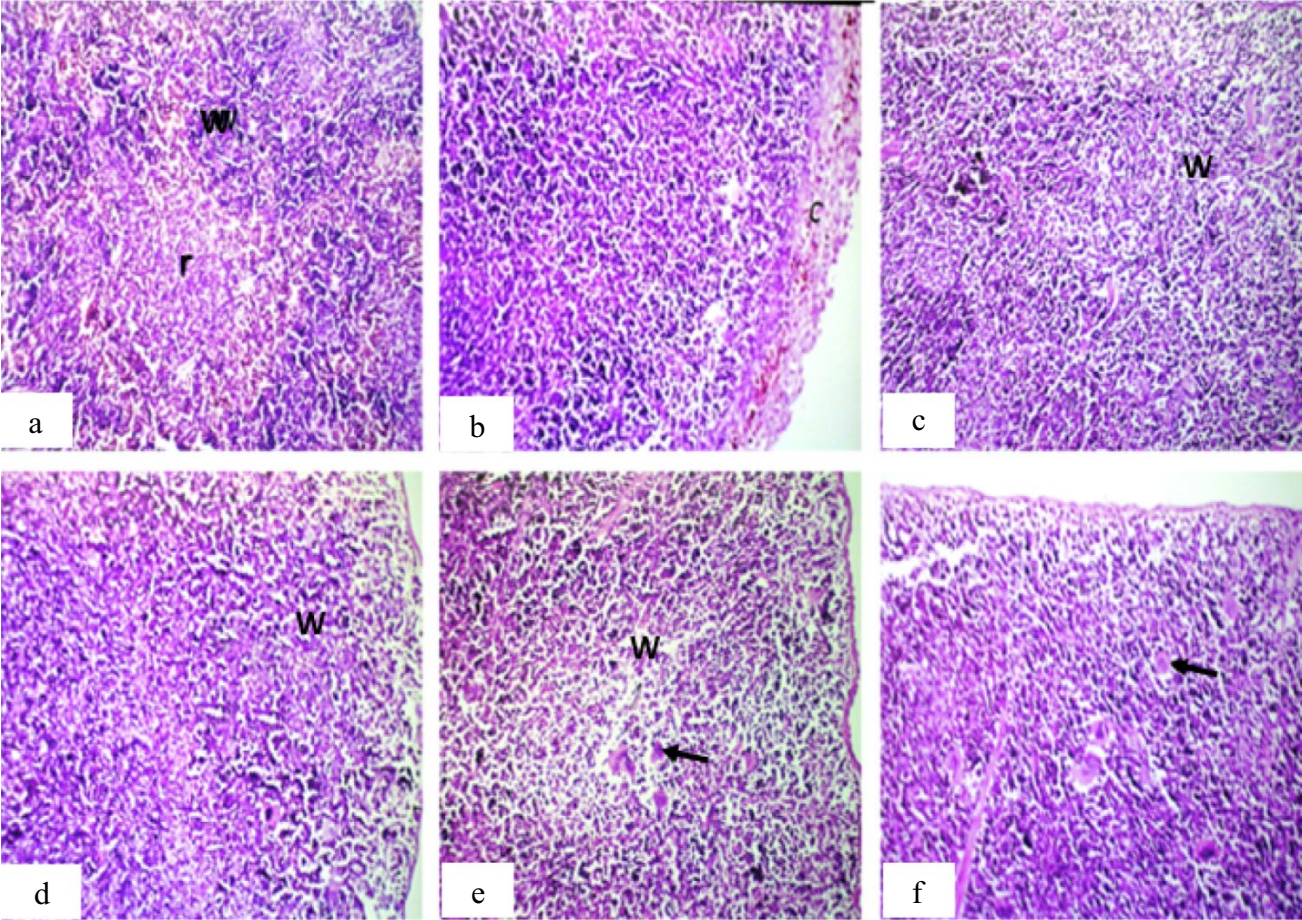
Photomicrograph of spleen sections of mice. **a** Group 1 (control) showing normal histological structure of the white (w) and red (r) pulps. **b** Group 2 (IP-MNPs) showing thickening, pigmentation, and inflammatory cell infiltration in the splenic capsule (c). **c** and **d** group 3 (IM-MNPs) and group 4 (ESC) showing lymphoid depletion in the white pulps (w). **e** Group 5 (ESC + IP-MNPs) showing lymphoid depletion in the white pulps (w) with massive number of megakaryoblasts (arrow). **f** Group 6 (ESC + IT-MNPs) showing massive number of megakaryoblasts (arrow). H&E, 40 ×

For the spleen, the main observation was found in production of massive number of megakaryoblasts in the tumor groups, while the other treated groups show inflammatory cell infiltration in the splenic capsule and lymphoid depletion in the white pulps (Fig. 6).

**Fig. 7:**
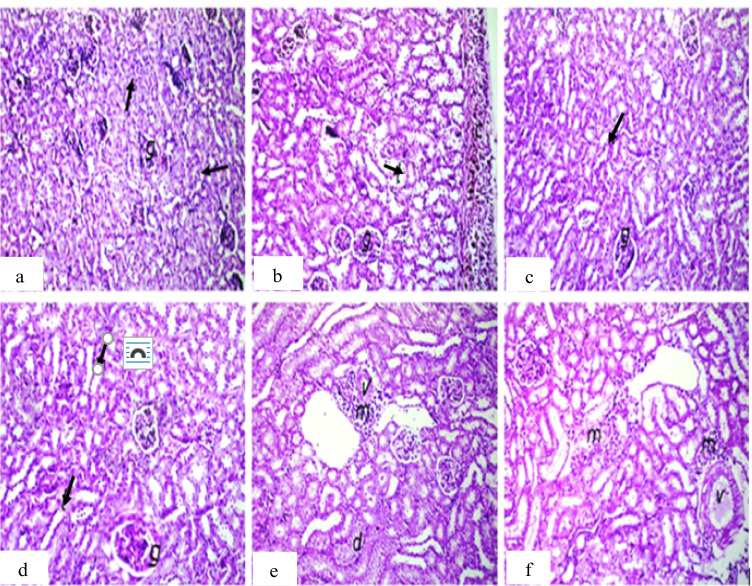
Photomicrograph of kidney sections of mice. **a** Group 1 (control) showing normal histological structure of the glomeruli (g) and tubules (arrow) at the cortex. **b** Group 2 (IP-MNPs) showing thickening, pigmentation, and inflammatory cell infiltration in the renal capsule (c). **c**, **d** Group 3 (IM-MNPs) and group 4 (ESC) showing normal histological structure. **e** Group 5 (ESC + IP-MNPs) showing perivascular inflammatory cell infiltration (m) (v = vein) with degeneration in the lining epithelium of tubules (d). **f** Group 6 (ESC + IT-MNPs) showing focal inflammatory cell infiltration in between the tubules and in the perivascular area (m) (v = vein). H&E, 40 ×

For the kidney, the main observation was inflammatory cell infiltration in the renal capsule or between the renal tubules in all treated groups except IM injected groups (Fig. 7).

**Fig. 8:**
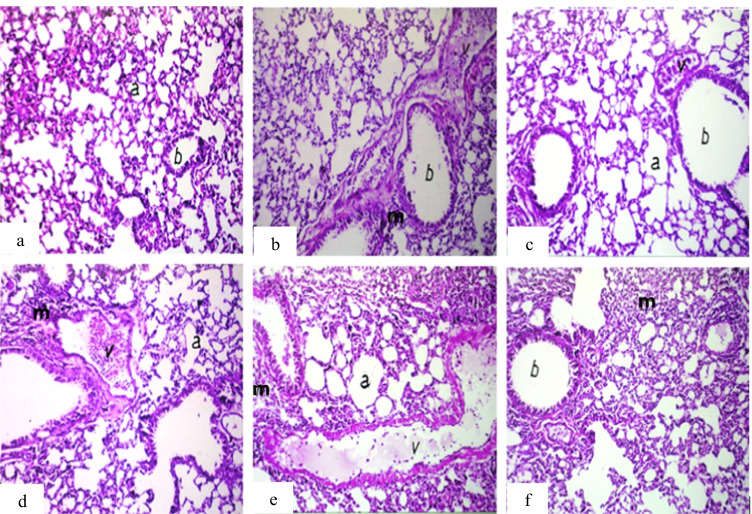
Photomicrograph of lung sections of mice. **a** Group 1 (control) showing normal histological structure of the alveoli (a) and bronchioles (b). **b** Group 2 (IP-MNPs) showing peribronchiolar inflammatory cell infiltration (m). **c** Group 3 (IM-MNPs) showing emphysema in air alveoli (a) with congestion in peribronchiolar blood vessels (v). **d** Group 4 (ESC) showing dilatation of the blood vessels (v) with perivascular inflammatory cell infiltration (m) and emphysema in the air alveoli (a). **e** Group 5 (ESC + IP-MNPs) showing dilatation and congestion of the blood vessels (v) with emphysema in the air alveoli (a) and inflammatory cell infiltration in between (m). **f** Group 6 (ESC + IT-MNPs) showing diffuse inflammatory cell infiltration in the air alveoli (m). H&E, 40 ×

For the lung, the main observation was peribronchiolar or perivascular inflammatory cell infiltration in all treated groups compared to the control group (Fig. 8).

### MNP-Induced DNA Damage in ESC and Liver Tissues

**Fig. 9:**
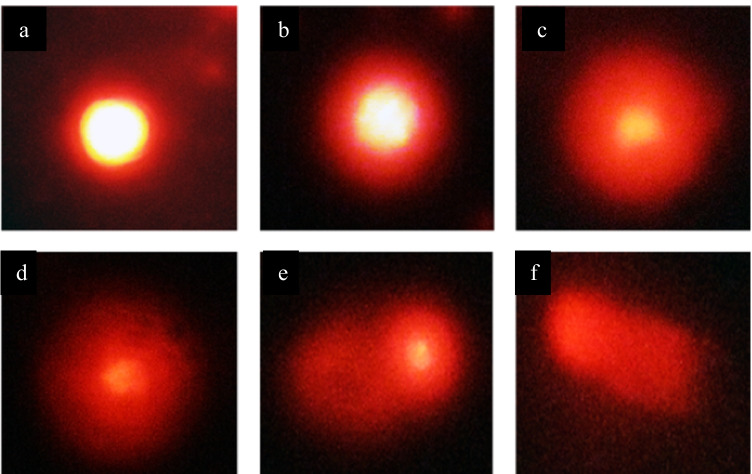
Photomicrograph showing typical nuclei with various degrees of DNA damage observed as comet. All types of comets are seen in all studied groups, but the frequency of each type differed between normal control and treated groups with MNPs. 400 ×

Genotoxicity study using comet assay was performed on ESC, skeletal muscles, liver, and brain tissues. The extent of DNA damage for all samples was evaluated using DNA percentage in tail that represents the intensity of all tail pixels divided by the total intensity of all pixels in the comet. Figure 9 shows typical nuclei with various degrees of DNA damage that were observed as comet.

**Fig. 10:**
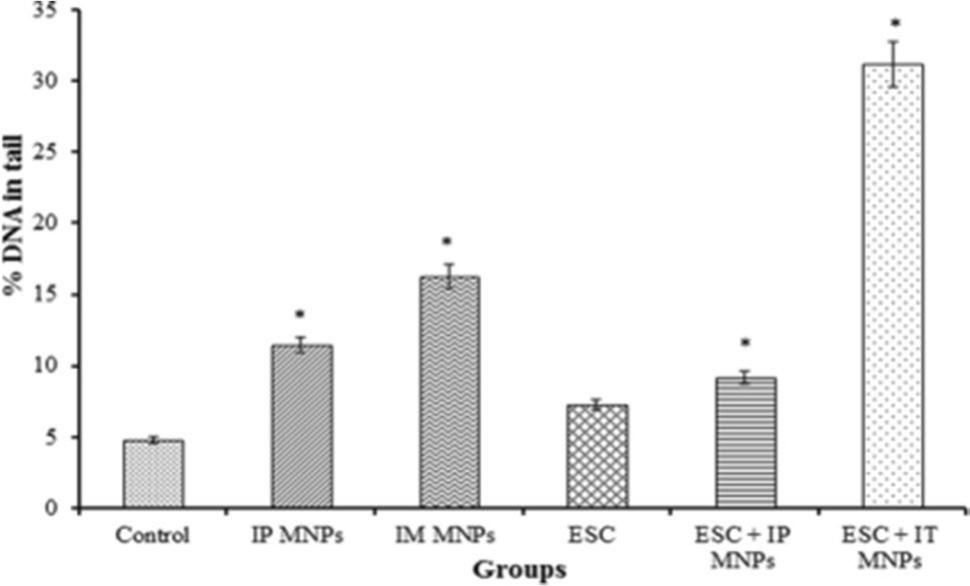
Effect of MNP injection on DNA damage induced in solid Ehrlich tumor and muscle tissues. *P < 0.05 vs non-treated control

By comparing the percentage of DNA in tail that was observed in ESC and skeletal muscles, treatment with MNPs resulted in significant increase in percentage of DNA in comet tail, indicating induction of DNA damage. As shown in Table 2 and Fig. 10, all treated groups have statistically significant increase (*P* < 0.05) in percentage of DNA in tail compared with the control group. Also, it was observed that more DNA damage was induced by local injection of IM treated with MNPs or IT treated with MNPs than IP injection in IP treated mice and IP treated ESC.

**Table 2:** Effect of MNP injection on DNA damage induced in ESC and muscle tissues

Groups	Tail length (µm) (mean ± SE)	% DNA in tail (mean ± SE)	Tail moment (µm) (mean ± SE)
Group 1 (control) (muscles)	5.7 ± 1.4 ^a^	4.8 ± 0.5 ^a^	0.3 ± 0.6 ^a^
Group 2 (IP MNPs) (muscles)	12.9 ± 2.1 ^b, c,*^	11.5 ± 1.8 ^b,*^	1.5 ± 2.0 ^b,*^
Group 3 (IM MNPs) (muscles)	13.1 ± 1.7 ^c,*^	16.3 ± 1.4 ^c,*^	6.2 ± 0.7 ^c,*^
Group 4 (ESC) (tumor)	11.2 ± 1.8 ^b, c,*^	7.3 ± 0.6 ^a, b,*^	0.8 ± 0.9 ^a,b, c^
Group 5 (ESC + IP MNPs) (tumor)	9.15 ± 1.0 ^a,b^	9.14 ± 1.2 ^a, b,*^	0.9 ± 0.2 ^a,b^
Group 6 (ESC + IT MNPs) (tumor)	20.8 ± 0.9 ^c,*^	31.2 ± 3.2 ^d,*^	11.2 ± 1.1^c,*^

All values are represented as mean ± standard error of mean (SE). There is a significant difference between groups by using one-way ANOVA at *P* < 0.05 followed by Duncan multiple comparison test. The same letter means that there is no significant difference between the two groups by using Duncan multiple comparison test (*P* > 0.05). The different letters mean that there is a significant difference between the two groups by using Duncan multiple comparison test (*P* < 0.05). *Statistically significant compared with negative control using Student’s *t*- test

**Fig. 11:**
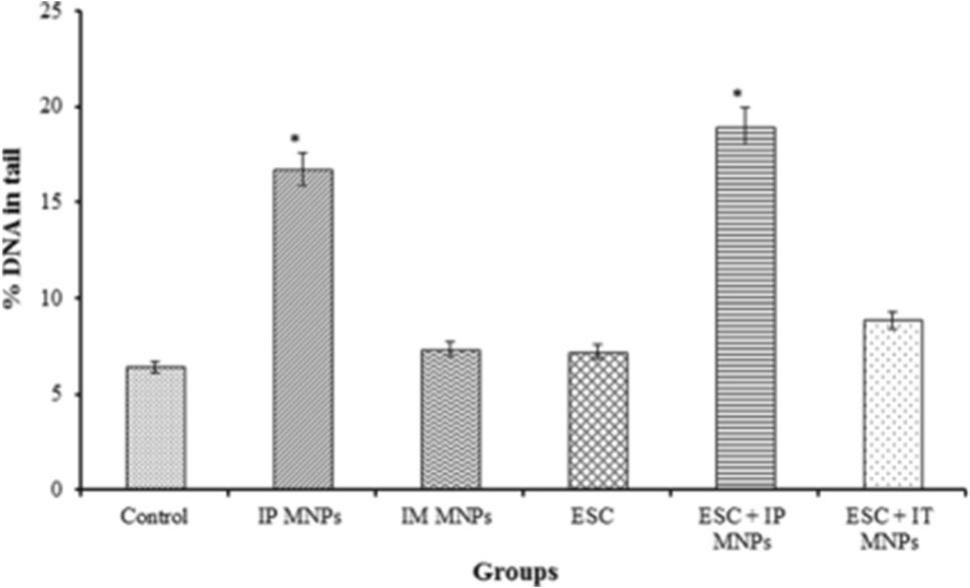
Effect of MNP injection on DNA damage induced in liver tissues. *P < 0.05 vs non-treated control

By comparing the percentage of DNA in comet tail in liver tissues among groups, a statistically significant difference was found in percentage of DNA in comet tail in the group treated IP with MNPs and ESC treated IP with MNPs (16.7 ± 0.9 and 18.9 ± 1.2, respectively) (*P* < 0.05) compared to control (Table 3, Fig. 11).

**Table 3:** Effect of MNP injection on DNA damage induced in liver tissues

Groups	Tail length (µm) (mean ± SE)	% DNA in tail (mean ± SE)	Tail moment (µm) (mean ± SE)
Group 1			
(control)	4.2 ± 0.7 ^a^	6.4 ± 0.8 ^a^	0.3 ± 0.5 ^a^
Group 2			
(IP MNPs)	13.7 ± 1.4 ^c,*^	16.7 ± 0.9 ^b,*^	2.3 ± 1.3 ^b^
Group 3			
(IM MNPs)	8.4 ± 2.6 ^a,b^	7.4 ± 1.1 ^a^	0.7 ± 0.9 ^a^
Group 4			
(ESC)	6.4 ± 0.8 ^a,b^	7.2 ± 0.2 ^a^	0.5 ± 0.3 ^a^
Group 5			
(ESC + IP MNPs)	7.9 ± 2.3 ^a,b^	18.9 ± 1.2 ^b,*^	1.5 ± 1.2 ^a^
Group 6			
(ESC + IT MNPs)	10.9 ± 1.1 ^b, c,*^	8.9 ± 1.0 ^a^	1.0 ± 0.3 ^a, b,*^

All values are represented as mean ± standard error of mean (SE). There is a significant difference between groups by using one-way ANOVA at *P* < 0.05 followed by Duncan multiple comparison test. The same letter means that there is no significant difference between the two groups by using Duncan multiple comparison test (*P* > 0.05). The different letters mean that there is a significant difference between the two groups by using Duncan multiple comparison test (*P* < 0.05). *Statistically significant difference compared to negative control using Student’s *t*-test

**Fig. 12:**
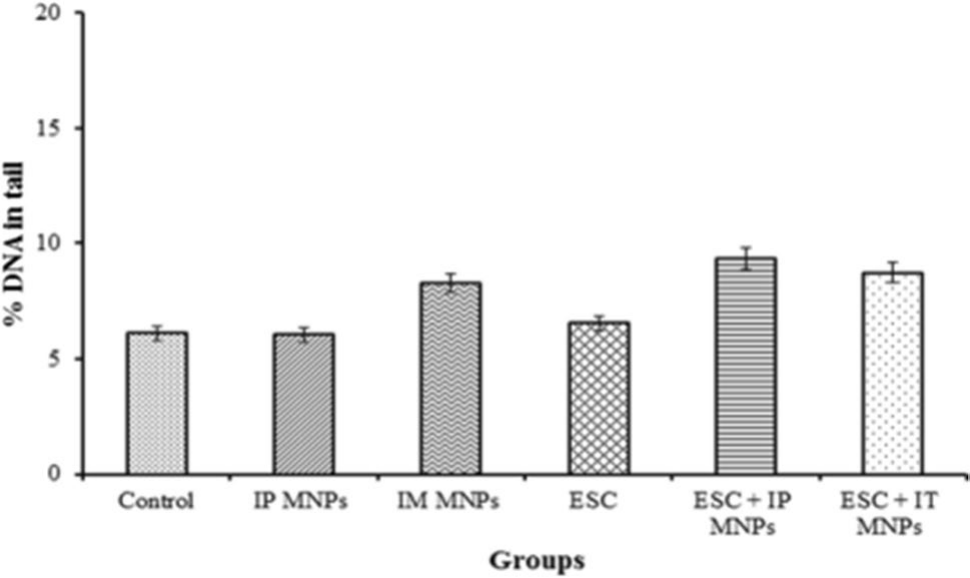
Effect of MNP injection on DNA damage induced in brain tissues

On the other hand, no statistically significant difference was found by comparing the percentage of DNA in comet tail in brain tissues among groups (*P* > 0.05). So MNPs may have no effect on DNA of brain tissue (Table 4, Fig. 12).

**Table 4:** Effect of MNP injection on DNA damage induced in brain tissues

Groups	Tail length (µm) (mean ± SE)	% DNA in tail (mean ± SE)	Tail moment (µm) (mean ± SE)
Group 1			
(control)	3.9 ± 0.9 ^b^	6.1 ± 0.8 ^a^	0.3 ± 0.4 ^a^
Group 2			
(IP MNPs)	4.9 ± 0.6 ^a, b^	6.05 ± 0.32 ^a^	0.3 ± 0.2 ^a^
Group 3			
(IM MNPs)	8.5 ± 1.9 ^a, b^	8.3 ± 1.9 ^a^	0.7 ± 0.2 ^a^
Group 4			
(ESC)	11.6 ± 2.2 ^a^	6.6 ± 0.3 ^a^	0.8 ± 1.2 ^a^
Group 5			
(ESC + IP MNPs)	8.4 ± 2.9 ^a,b^	9.3 ± 1.7 ^a^	0.8 ± 1.3 ^a^
Group 6			
(ESC + IT MNPs)	8.1 ± 1.9 ^a,b^	8.7 ± 0.2 ^a^	0.7 ± 0.7 ^a^

All values are represented as mean ± standard error of mean (SE). There is a significant difference between groups by using one-way ANOVA at *P* < 0.05 followed by Duncan multiple comparison test. The same letter means that there is no significant difference between the two groups by using Duncan multiple comparison test (*P* > 0.05). The different letters mean that there is a significant difference between the two groups by using Duncan multiple comparison test (*P* < 0.05)

## Discussion

Nanomaterials have attractive characteristic features than larger particles; they are small enough to cross biological barriers such as the blood vessel walls and cell membrane [33]. Most nanomaterials achieve selective tumor accumulation via the enhanced permeability and retention (EPR) effect or targeting to cellular receptors [34]. It was reported that toxic effects of NPs are controversial and may be dependent on particle size, surface coating, exposure route, and exposure duration [35]. Magnetite nanoparticles (MNPs) have promising approach in tumor detection, screening, and destruction [36, 37].

Regarding the dose, previous in vivo toxicological studies indicated no mortalities in mice treated with MNPs using up to 60 ppm, the same dose that was used in the present study and in an early study (patent co-author TAS) for treating anemia [24]. Previously, the first tolerance study with carbohydrate-coated magnetic nanoparticles as potential delivery systems was performed in nude mice and showed no median lethal dose (LD_50_) after injection of magnetic nanoparticles [38, 39].

Previous results of the authors showed that MNPs enhanced the expression of p53 and p16 genes, tumor suppressor genes, those direct cells to trigger programmed cell death by apoptosis in ESC cells [23].

Herein, after injection of control and tumor-bearing mice with the synthesized MNPs IP and IT, tumor and normal tissue distribution of MNPs was evaluated in normal and tumor-bearing mice. Furthermore, the histopathological effects and DNA damage induced by MNPs in normal and tumor tissues were investigated. This study will give an insight into the possible adverse effects of MNPs on different tissues and organs. It is important to know the biological responses to NPs and their safety when they are circulated, distributed, and accumulated in each tissue during the application. Histopathology studies conducted by different investigators showed that MNPs such as fluorodeoxy glucose–conjugated magnetite nanoparticles (FDG-mNPs) showed different distribution and histological changes in cancer-bearing mice [40]. The magnetic fibrin nanoparticles are highly accumulated in the tissues of the liver and spleen more than other organs as demonstrated by histopathological analysis [41].

Results here revealed that the liver, spleen, lung, and heart have significantly higher iron content in groups treated IP. While the tumor, muscles, and liver have significantly higher iron content in groups treated locally. Regarding to its effect on DNA, MNPs induced significant DNA damage in tumor injected locally. Significant DNA damage was induced in the liver in IP injected groups, but no significant DNA damage could be detected in the brain. Histopathological findings in ESC revealed marked tumor necrosis, 50% in IT injected group, 40% in IP injected group, and 20% only in untreated tumors. Other findings include inflammatory cell infiltration, dilatation, and congestion of blood vessels of different organs of treated groups in addition to appearance of metastatic cancer cells in the liver of non-treated tumor group.

During the experiment, lower activity and immobility of mice in tumor-bearing groups were observed due to development of solid tumor in the thigh. However, no other signs of toxicity were observed in all mice groups including weight loss, seizures, disheveled hair, irregular respiration, gastrointestinal symptoms, convulsions, severe decubitus paralysis, or death. Postmortem examination did not find significant changes in organ weights and morphology in all mice groups. The same observation was noticed in rat model administered with MNPs coated with meso-2, 3-dimercaptosuccinic acid (DMSA) and conjugated to PEG-derived molecules [29].

Herein, distribution of MNPs was detected by ICP measurements in various target organs and tissues. MNPs accumulated in tumor and muscle tissues comparably after IT and IM administration much more than after IP administration. The present results suggest that MNPs could successfully be uptaken by tumor tissues and more accumulated in tumor after IT injection, as a result of enhanced permeability and retention (EPR) in tumor tissue and the perforated leaky tumor-associated blood vessels which allow molecules to accumulate passively in the tumor microenvironment [42, 43]. Similarly, an earlier research showed that the concentrations of nanoparticles in cells affected by hematologic malignancy were much higher than those in normal somatocytes and can stay longer in blood [14, 44].

Furthermore, the liver, spleen, lung, and the heart showed significantly higher iron content in groups treated IP compared to control. Similar results have been observed for MNPs coated with PEG and dextran. Moreover, various studies have shown that MNPs have taken up by the reticuloendothelial system (RES) that is rich in macrophages including the liver (80–90%) and spleen (5–8%) [29, 45–49] more than in other organs including the brain, heart, kidney, and lungs [50, 51].

The liver showed much higher iron content than in tumor after IP injection which is similar to another study found that liver uptake for the MNPs is greater than for the tumor after intravenous injection [52]. On the other hand, groups treated locally have significantly higher iron content in the tumor, muscles, and liver with lower iron concentration in their other organs. This agrees with a previous study found that most of magnetic NPs, injected IT in mice model, remained in tumors and less than 1% of injected NPs were detected in the liver and spleen [49, 53]. Also, in a rat model of 9L-glioma, brain tumors administered PEG- magnetic NPs [47, 49]. It was reported that iron oxide nanoparticles ranging from 5 to 150 nm may offer the most effective distribution in certain tissues, especially in tumors [54, 55].

In the present study, groups of mice injected with Ehrlich tumor cells (4, 5, and 6) were injected with approximately the same number of cells (about 2 × 10^6^ cells) and showed the same tumor size at the beginning of experiment before treatment with MNPs without significant difference among groups. Interestingly, more necrotic effect of MNPs was observed on treated tumor compared to non-treated tumor. In addition, MNPs reduced metastasis in treated groups.

In the same context, a previous study reported a cell death and reduced proliferation of fibroblasts in vitro induced by uncoated MNPs and dextran-MNPs [11]. Also, uncoated SPIONS caused significant cell death in dermal fibroblasts, while lung cells were not affected [15, 56]. Another study found that magnetic iron oxide nanoparticles caused hyperthermia-mediated oncotic necrosis in head and neck cancer mouse model compared to non-treated tumors [13].

Histological examination was performed for injected sites and other organs (liver, spleen, kidney, lung, and brain) to look for signs of MNP accumulation and test their side effects on the other organs. It could be concluded that MNPs induced inflammatory responses with moderate toxicological effects in most of examined organs.

It is known that NPs can gain access to the nucleus and may induce DNA damage [57]. Herein, as detected by comet assay, MNPs induced a significant DNA damage in tumor injected locally. While a significant DNA damage was induced in the liver in groups that were injected IP, but no significant DNA damage was detected in the brain. Induced DNA damage may be attributed to free iron ions, those that catalyze the amplification of reactive oxygen species (ROS) and generation of highly reactive radicals through Fenton reactions [52]. MNPs coated with polyaspartic acid caused increase in micronucleus frequency in Swiss mice [21]. As well as MNPs caused high level of oxidative DNA lesions in A549 human lung epithelial cell line [58]. Furthermore, a concentration-dependent DNA damage was observed in SPION-treated L-929 fibroblasts cells [59]. Another study detected DNA damage induced by MNPs in Salmonella strains TA100, TA2638, TA102, and TA98 [60]. Cytotoxicity of superparamagnetic iron oxide nanoparticles was emphasized at several levels, including cellular changes such as oxidative stress-induced damage to nucleic acids and altered cellular responsiveness [61].

## Conclusion

In conclusion, the present study has shown that MNPs may have antitumor effect on ESC grown subcutaneously in Swiss albino mice. Moreover, IT injection of MNPs is preferable to direct these NPs to tumor tissues and avoid their side effects on the normal tissues. This might be a good approach that opens a gate for new drug modality using nanotechnology.

## Data Availability

The datasets used and/or analyzed during the current study are available from the corresponding author on reasonable request.
